# Dominant phytoplankton groups as the major source of polyunsaturated fatty acids for hilsa (*Tenualosa ilisha*) in the Meghna estuary Bangladesh

**DOI:** 10.1038/s41598-022-24500-2

**Published:** 2022-12-05

**Authors:** Dinesh Chandra Shaha, Jahid Hasan, Sampa Rani Kundu, Fatimah Md. Yusoff, Mohammad Abdus Salam, Murshida Khan, Farhana Haque, Minhaz Ahmed, Mohammad Jalilur Rahman, Md. Abdul Wahab

**Affiliations:** 1 Coastal and Marine Dynamics Laboratory, Department of Fisheries Management, Faculty of Fisheries, Bangabandhu Sheikh Mujibur Rahman Agricultural University, Gazipur, 1706 Bangladesh; 2 National Oceanographic and Maritime Institute, Dhaka, Bangladesh; 3 International Institute of Aquaculture and Aquatic Sciences, Universiti of Putra Malaysia (UPM), 71050 Port Dickson, Negeri Sembilan Malaysia; 4 Department of Genetics and Fish Breeding, Bangabandhu Sheikh Mujibur Rahman Agricultural University, Gazipur, 1706 Bangladesh; 5 Department of Fisheries Technology, Bangabandhu Sheikh Mujibur Rahman Agricultural University, Gazipur, 1706 Bangladesh; 6 Department of Agroforestry and Environment, Bangabandhu Sheikh Mujibur Rahman Agricultural University, Gazipur, 1706 Bangladesh; 7 WorldFish, Bangladesh and South Asia Office, House 42/A, Road 114, Gulshan 2, Dhaka, 1212 Bangladesh

**Keywords:** Ecology, Environmental sciences, Ocean sciences

## Abstract

The tropical estuarine ecosystem is fascinating for studying the dynamics of water quality and phytoplankton diversity due to its frequently changing hydrological conditions. Most importantly, phytoplankton is the main supplier of ω3 polyunsaturated fatty acids (PUFA) in the coastal food web for fish as they could not synthesize PUFA. This study evaluated seasonal variations of water quality parameters in the Meghna River estuary (MRE), explored how phytoplankton diversity changes according to hydro-chemical parameters, and identified the major phytoplankton groups as the main source of PUFA for hilsa fish. Ten water quality indicators including temperature, dissolved oxygen, pH, salinity, dissolved inorganic nitrogen (DIN = nitrate, nitrite, ammonia) and phosphorus, dissolved silica and chlorophyll-a were evaluated. In addition, phytoplankton diversity was assessed in the water and hilsa fish gut. Principal component analysis (PCA) was used to analyze the spatio-temporal changes in the water quality conditions, and the driving factors in the MRE. Four main components were extracted and explained 75.4% variability of water quality parameters. The most relevant driving factors were dissolved oxygen, salinity, temperature, and DIN (nitrate, nitrite and ammonia). These variabilities in physicochemical parameters and dissolved inorganic nutrients caused seasonal variations in two major groups of phytoplankton. Peak abundance of Chlorophyta (green algae) occurred in water in nutrient-rich environments (nitrogen and phosphorus) during the wet (36%) season, while Bacillariophyta (diatoms) were dominant during the dry (32%) season that depleted dissolved silica. Thus, the decrease of green algae and the increase of diatoms in the dry season indicated the potential link to seasonal changes of hydro-chemical parameters. The green algae (53.7%) were the dominant phytoplankton group in the hilsa gut content followed by diatoms (22.6%) and both are contributing as the major source of PUFAs for hilsa fish according to the electivity index as they contain the highest amounts of PUFAs (60 and 28% respectively).

## Introduction

An estuary is a semi-enclosed body of water with open or intermittent connections to the sea^1^. Biophysical and chemical components in a healthy estuary persist within the limits of natural change. The growth rate and dominance of the estuarine phytoplankton, which forms an important food item for hilsa (*Tenualosa ilisha*) are influenced by the changes in the physicochemical parameters^2^. Although these parameters vary, they are strongly influenced by local weather and climate change and can be interpreted as seasonal characteristics^3^. Therefore, studying the interaction between water quality and phytoplankton diversity of tropical estuarine ecosystems due to frequently changing hydrological conditions is very important.

Seasonality determines the variation of physicochemical parameters such as salinity, temperature, pH, nitrate, nitrite, ammonia, silicate and inorganic phosphate, which in turn affect the species composition and diversity of the phytoplankton community in the estuarine ecosystem^4^. Generally, local rainfall, tidal inflow, and several abiotic and biotic processes play a significant role in temporal fluctuations of the nutrient cycle in estuaries^5^. The important macronutrients for most phytoplankton species are nitrate and phosphate, although diatoms additionally need silicate to construct their frustules. However, each phytoplankton species has its own favourable environmental conditions for multiplication^6^. For example, Chlorophyta (green algae) proliferate rapidly in a nutrient rich (especially nitrogen and phosphorus) environment with favourable temperature (> 25 °C)^7^. Gamier, et al.^8^ reported that low nitrogen conditions usually limit the reproduction of Chlorophyta species. On the other hand, Cyanobacteria are typically dominant in the low salinity estuarine zone^9–11^. In fact, most cyanobacteria are freshwater species. In addition, silica is taken into account as a primary controlling factor of the diatom-green algae succession because its availability is of vital importance for the occurrence of diatoms. Thus, the local processes related to the physicochemical parameters lead to the pattern of phytoplankton diversity.

The large Meghna River estuarine (MRE) system serves as an important spawning ground for hilsa fish (*Tenualosa ilisha*) in favourable environmental conditions^12,13^. For example, hilsa prefers freshwater (salinity < 0.1 PSU) for spawning and nursery activities^14,15^. Although some research has been carried out on the biophysical assessments and phytoplankton diversity of the MRE^16,17^, the effects of abiotic parameters on phytoplankton communities have not been studied. In addition, hilsa is the best source of ω3 polyunsaturated fatty acids (PUFAs) for human consumption^18^, and the primary food source is the phytoplankton^19^. But fish or crustaceans cannot readily biosynthesize the ω3 and ω6 polyunsaturated fatty acids (PUFAs), and have to obtain them from their diet such as phytoplankton^19^. The highest proportion of PUFA is found in green algae, with approximately 60% of the total fatty acids^19^. In contrast, the lowest PUFA is found in blue-green algae (Cyanobacteria) and diatoms (26 and 28% respectively)^19^. However, information on the major phytoplankton groups having significant contribution to the supply of PUFA to hilsa fish is scarce. The purpose of this study was to (i) to evaluate the spatial and seasonal variation of major water quality parameter in the MRE using multivariate statistical techniques, (ii) to explore how phytoplankton diversity changes with changing hydro-chemical parameters and (iii) to identify the major phytoplankton groups as main source of ω3 polyunsaturated fatty acids (PUFA) for hilsa fish. In this study, an effort has been made to establish link between variations in abiotic parameters influencing water quality and the phytoplanktonic diversity of the MRE, and identify major phytoplankton groups for PUFA.

## Material and methods

### Study area

**Figure 1:**
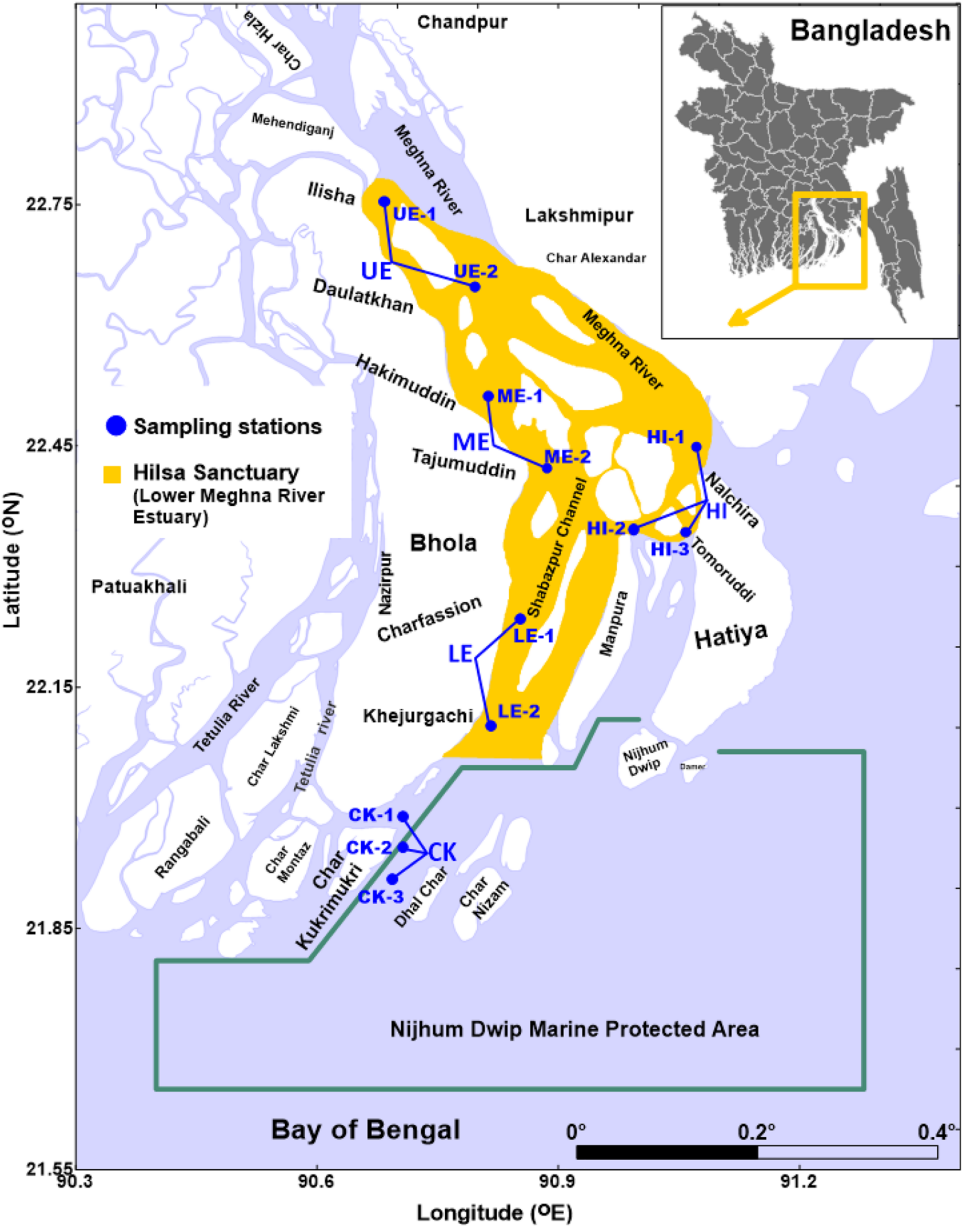
Map of the study area in the Meghna River estuary and its adjacent coastal area. The map was generated using QGIS (version 3.2.1, https://www.qgis.org). Conductivity-temperature-depth (CTD) recorder and water samples collection stations shown as solid circles.

The Meghna River system is the third largest freshwater outlet in the world (Fig. 1). The Meghna River brings huge river discharge of ∼1.5 × 10^12^ m^3^ year^−1^ into the Bay of Bengal. A maximum discharge of approximately 82,000 m^3^ s^−1^ occur in the wet season and a minimum of < 10,000 m^3^ s^−1^ in the dry season. An annual average of approximately is 32,000 m^3^ s^−1^. Huge river discharge and rainfall during the wet season mainly regulate water temperature, salinity, nutrients export and primary productivity of the Meghna River basin. Otero et al.^20^ revealed that salinity distribution is mainly controlled by river discharge and other atmospheric variable like local rainfall. The presence of marine–brackish–freshwater ecosystems controlled by monsoon river discharge and tide greatly support hilsa fishery in the coastal waters of Bangladesh. The present study was carried out in the large Meghna River estuarine system and its adjacent coastal waters (Fig. 1).

### Sampling design

In this study, we established five sections (12 sampling sites) in the upper, middle, lower and sea-side of the Meghna River estuary (Fig. 1). In the sea-side section (OE), three sampling sites were selected at Char Kukrimukri (CK, Longitude: 90.7085, Latitude: 21.9528) and Hatiya Island (HI, Longitude: 91.0824, Latitude: 22.3826). In the upper, middle and lower sections, two sampling sites were selected at Ilisha (UE, Longitude: 90.6922, Latitude: 22.6788), Hakimuddin (ME, Longitude: 90.8191, Latitude: 22.4514) and Charfasson (LE, 90.7962, 22.1851). The along components of the study area are the riverine (UE) and estuarine zone (ME, LE) with six sampling sites and thence the sampling transects extend to the sea-side (CK and HI) of the MRE with six sampling sites. Vertical salinity, water and phytoplankton samples were collected in February, April, July, August, October and December in 2020. In addition, vertical salinity profiles were measured in January, March and June in 2021. Sample collections were conducted under low flow (January, February, March and December: dry season) and high flow (July, August and October: wet season) conditions at twelve sites in the Meghna River estuary.

### In situ measurements of water quality parameter

The water quality parameter (such as salinity, temperature, DO etc.) were measured with a conductivity-temperature-depth (CTD) profiler (model: In-situ Aqua TROLL 500, In-situ Inc., USA) in the mouth of the lower Meghna River estuary and its adjacent coastal area (Fig. 1). Speed boats or mechanized boats were used for in situ measurement and to collect water samples. Global positioning system (GPS) was used to collect samples from the accurate sampling stations.

### Dissolved inorganic nutrients

Water samples were collected at depths of 0–0.5 m with a water sampler of 1.5 L (Wildco Instruments, USA). Water samples were filtered through a fibreglass filters using a vacuum system and Whatman GF/C filter papers of porosity about 0.45 μm Millipore HA. After filtering, the filtrates were stored in the refrigerator until analyses. The concentration of dissolved inorganic nutrients [Nitrate–N (NO_3_–N), Nitrite–N (NO_2_–N), ammonia (NH_4_^+^), orthophosphates (PO_4_-P) and dissolved silicon compounds—Dsi] were analysed with standard spectrophotometric methods^21^. Spectrophotometer (Model: DR6000 HACH, USA) was used to measure absorbance. The USEPA Ascorbic acid method was used for phosphorus (PO_4_^3−^), the USEPA Nessler method for ammonium (NH_4_^+^)^22,23^, and the reduced copper cadmium method^22^ for total oxidised nitrogen (NO_3_-N and NO_2_-N). Inorganic nutrients include both dissolved inorganic nitrogen (DIN ≈ NH_4_^+^, NO_3_-N and NO_2_-N) and phosphorus (DIP ≈ PO_4_-P).

### Primary producers

#### Phytoplankton biomass

Water samples were collected to determine phytoplankton biomass (measured as chlorophyll-a concentrations) of the MRE. Chlorophyll-a pigment was extracted by filtering 1 L of water through a vacuum machine using Whatman GF/C filter papers of porosity about 0.45 μm Millipore HA. Immediately after completion of filtration, the filters were placed into glass vials containing 10 ml of 95% ethanol (Merck 4111) for 24 h in a refrigerator for extracting chlorophyll-a pigment. Afterwards, pigment extraction was performed by gentle grounding with a homogenizer to speed up the extraction. After homogenizing, the extract was poured into a centrifuge tube and add acetone solution to make the volume up to 10 ml. The solution was centrifuged at 3000 rpm for 10 min. The supernatant solution was measured spectrophotometrically for pigment concentration (DR 6000, USA). The chlorophyll-a concentration was then determined using the SCOR-UNESCO^23^ equations for each sample.

#### Phytoplankton community composition

Phytoplankton samples were collected by towing phytoplankton net of mesh size of 20 μm horizontally. The concentrated water samples were then transferred into 15 ml plastic vials and added 10% buffered formalin to preserve in the refrigerator. Thereafter, qualitative analysis of phytoplankton samples was accomplished under a phase-contrast microscope (Primo Star, Carl Zeiss) for the taxonomic rank. For quantitative analysis, Sedgwick Rafter chamber (Wildlife, USA) was used for counting phytoplankton cells. The cells were classified according to different functional groups of algae, i.e. Bacillariophyta (diatoms), Miozoa (dinoflagellates), Cyanobacteria (blue-green algae) and Chlorophyta (green algae). The number of phytoplankton (cells L^−1^) was computed for each group using the equation defined by Snow et al.^24^.

#### Species diversity indices

The species diversity of a habitat is calculated using diversity indices. Phytoplankton diversity indices^9^ were calculated using the Simpson Diversity Index (D) and Simpson Reciprocal Index (1/D). Simpson Index varies from 0 to 1. Zero denotes a high diversity, while 1 represents a less diverse region^6^. Simpsons Reciprocal Index is proportionally related to species diversity.

$${\mathrm{Simpson\, index}} (D)=\sum n(n-1)/N(N-1)$$

where N is the total number of organisms of all species in an area; n is the total number of organisms of a particular species.

#### Collection of fish specimens and gut content analysis

Hilsa fish specimens of different sizes were collected randomly from fishermen of the Meghna River estuary. The freshly caught fish specimens were preserved in an insulated box with ice and transported to the laboratory. Ninety fish specimens were taken for gut content analysis. The length varied from 18 to 35 cm and the weight from 109 to 810 g. The alimentary canals from the oesophagus to the anus of the preserved hilsa fish were dissected and preserved in 10% buffered formalin. The gut contents from the stomach to the gizzard of the hilsa fish were then dissolved in water. Thereafter, available food organisms (phytoplankton ) were examined using an electrical microscope (Model: Carl Zeiss, Primo Star, Germany) and took pictures with photogenic devices for qualitative analysis. For the qualitative analysis, phytoplankton were then identified up to the genus level using the keys of Ward and Whipple^25–27^.

#### Electivity index

Suppose the predator is foraging in an environment (such as water) where the preys consist of two or more environmental prey taxa. Probability ${a}_{i}$ is a randomly selected environmental prey item belongs to taxon *i*, which we refer to as the “target” taxon for the analysis^28^. Also assume that the predator ate M prey and let ${g}_{i}$ (gut phytoplankton -) denote the probability that a randomly selected prey from M belongs to taxon *i.*

The electivity index was calculated from the odds ratio by a logistic transformation,

$${X}_{i}=\frac{{O}_{i}}{{1+O}_{i}}$$

$${\mathrm{where}}\; {\mathrm{O}}_{i}=\frac{{g}_{i}}{{1-g}_{i}}\times \frac{{1-a}_{i}}{{a}_{i}}$$

The index ${X}_{i}$ scales from 0 to 1. The value is 0.5 when the odds ratio is 1, indicating that the fraction of prey (species *i*) is the same for the environmental (water) prey sample and the gut sample^28^. The range should be at its maximum when g_i_ = 1 and minimum when g_i_ = 0. Most indices follow this criterion.

### Statistical analysis

The R version 4.0.3^29^ was used to perform the multivariate statistical analysis of spatiotemporal variations in the Meghna estuarine habitats. In the present study, 10 physico-chemical factors were used for the multivariate statistical analysis, including water temperature, dissolved oxygen, salinity, pH, chlorophyll-a, nitrate–N, nitrite-N, ammonia, phosphate-p, and dissolved silica. Descriptive statistics were determined for all of the physico-chemical and nutrient variables. As a complement, boxplot analysis was performed by using the ‘ggboxplot’ package. The paired samples Wilcoxon test is a non-parametric substitute to paired t-test used to compare between dry and wet seasons data. In contrast, Kruskal**–**Wallis test, an alternative of one-way ANOVA, is a non**-**parametric test used to compare the spatial variations. The Wilcoxon and Kruskal**–**Wallis tests were made using the ‘ggplot2’ package. The principal component analysis (PCA) was performed to relate the environmental factors (physico-chemical, dissolved nutrients and chlorophyll-a). The correlation matrix and the factorial axes analysed using PCA were showed significantly higher eigenvalues compared to those produced by matrices of the same dimension^30^. To confirm the presence of spatiotemporal variation among environmental factors along and across the MRE, PCAs were executed by using the ‘FactoMineR’ package using Euclidean distance method^30,31^. Furthermore, the contributions of the variables to the principal components (PCs) were observed to identify which environmental parameter were greatly differed among the different compartments of the MRE habitats. The four PCs (Dim1 ~ 4) were considered in this study to describe most of the variability. All the PCA were made using the ‘ggplot2’ package^32^. The correlations among the environmental factors were tested and plotted using the “Performance Analytics” packages^33^.

## Results

### Spatial and temporal variation of vertical salinity

**Figure 2:**
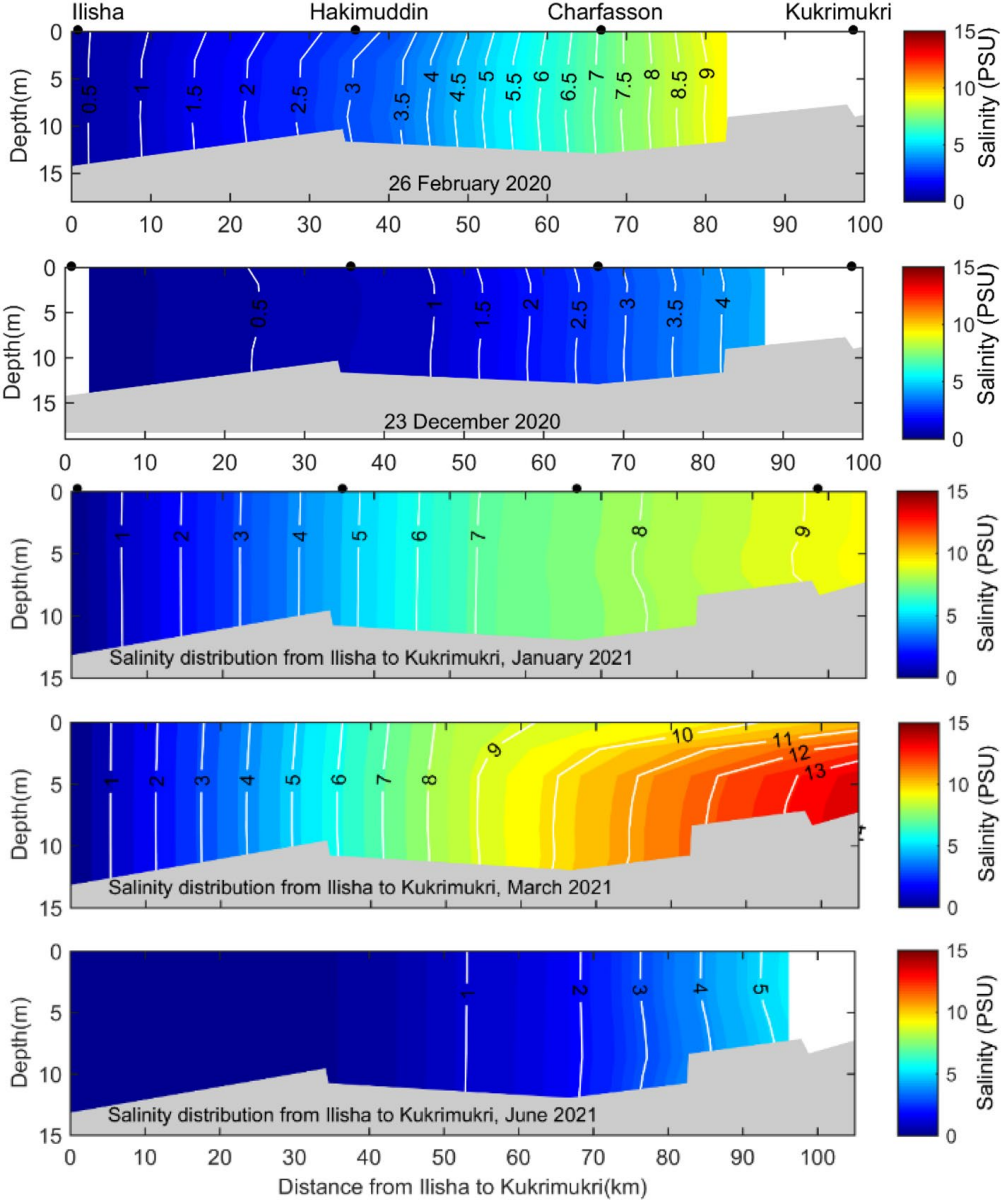
Vertical salinity distribution in the Lower Meghna River estuary during the dry (February, December, January, March and June) season in 2020 and 2021.

**Figure 3:**
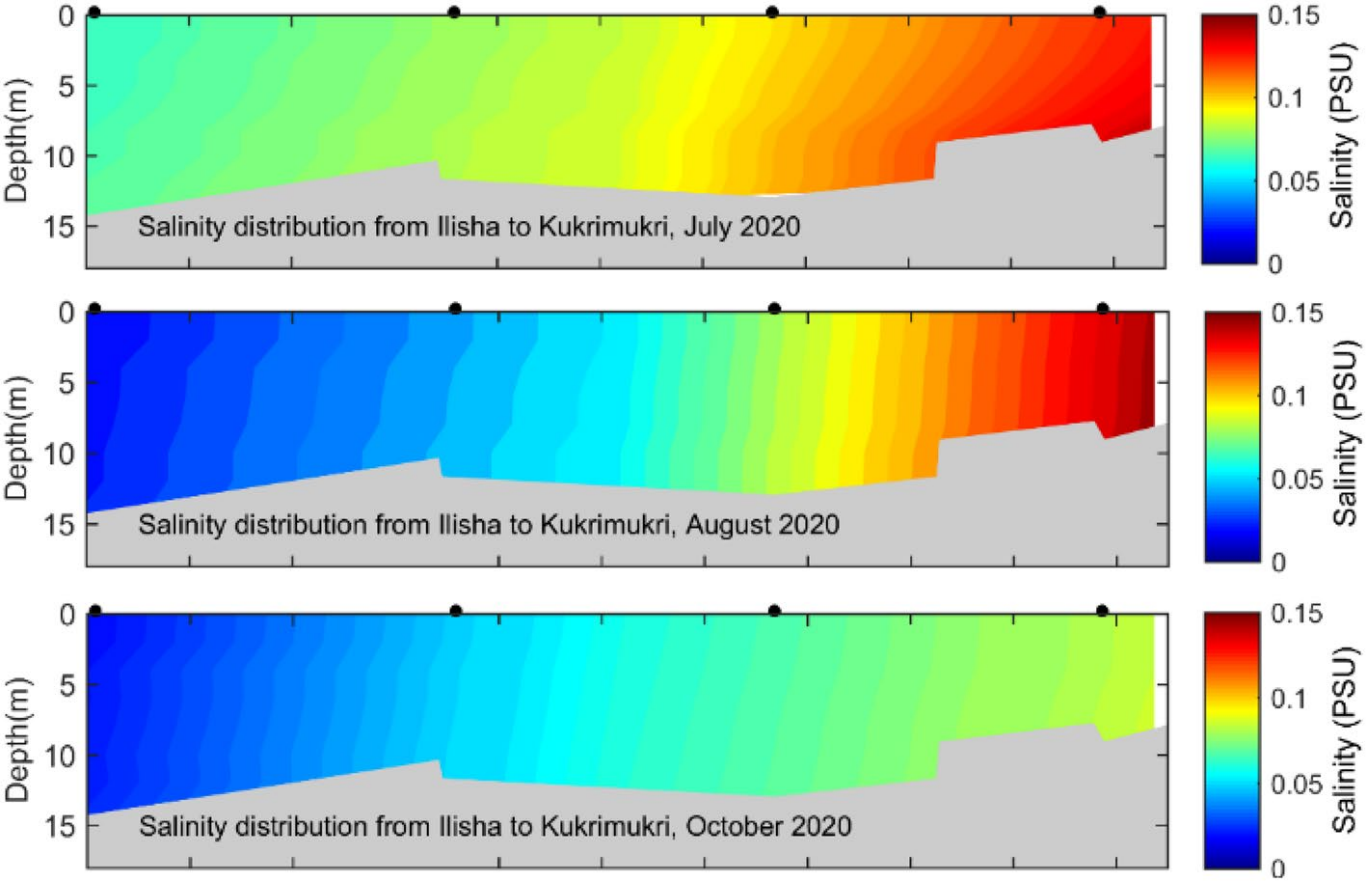
Vertical salinity distribution in the Lower Meghna River estuary during the wet (July, August and October) season in 2020 and 2021.

Salinity is a useful indicator to understand the hydrodynamic parameters of estuaries, including stratification^34,35^, flushing^36^, the distribution patterns of ecological parameters^37^. Water column stratification was assessed using the stratification parameter (${n}_{s}=\partial S/{s}_{m}^{\prime}$ where $\partial S$ = S_bot_-S_sur_, S_m_ = 1/2(S_bot_-S_sur_), with S_sur_ is the salinity at the surface and and S_bot_ the salinity at the bottom of the water column. The water column is well mixed when ${n}_{s}$ is < 0.1, partially mixed when 0.1< ${n}_{s}$  < 1.0 and stratified when ${n}_{s}$ > 1.0 ^38^. According to the stratification parameter (< 0.01), the MRE is a well-mixed estuary during the dry and wet seasons. The MRE can be characterized as a macrotidal estuary based on the tidal range criterion^39^, resulting the distribution of ecological parameters is homogeneous. The tidal range supports the well mixed condition of the MRE. In addition, the spatial distribution of salinity in the MRE showed the expected variations related to the annual rainfall regime and tide (Fig. 2). During the dry season, the maximum salinity value was 13 PSU at the downstream region, approximately 100 km seawards from the Ilisha ghat and the minimum salinity was < 0.15 PSU at the upstream region (Fig. 2). During the wet season, the salinity decreased to < 0.15 along the MRE. The salinity difference between the dry and wet seasons was 13 PSU in the MRE. The saline water persists for several months (December -June) in the MRE during the dry season (Fig. 2 and 3). By contrast, the saline waters of the MRE retreated to the coastal area during the wet season (July–October) due to increasing freshwater discharge from upstream and the MRE became fresh condition. The salinity section recorded in December showed a transition period for the MRE reversal from a freshwater system to a brackish water system (Fig. 2). In contrast, the June salinity section showed a transition period from the brackish water to freshwater system as the river discharge decreased (Fig. 3). The position of a near-bottom isohaline (2 PSU) along the MRE depends primarily on freshwater discharge and secondarily on tide. Hilsa shad prefers freshwater of < 0.1 PSU for spawning, 0–1 PSU estuarine water for nursing of the juveniles and 0–2 PSU estuarine and coastal water for brood fish^14^. Thus, the temporal variation in vertical salinity profiles indicated that the MRE is a suitable hilsa spawning and nursery habitat during the wet season (Fig. 3). However, hilsa spawn all the year round with a major spawning season during the wet season (September–October) under the full moon phase^40^. In contrast, during the dry season, the upper MRE (above Ilisha ghat, UE) will act as suitable hilsa spawning and nursery habitat all the year round as per vertical salinity distribution of the MRE.

### Seasonal and spatial variation of water quality

#### Physicochemical parameters

**Figure 4:**
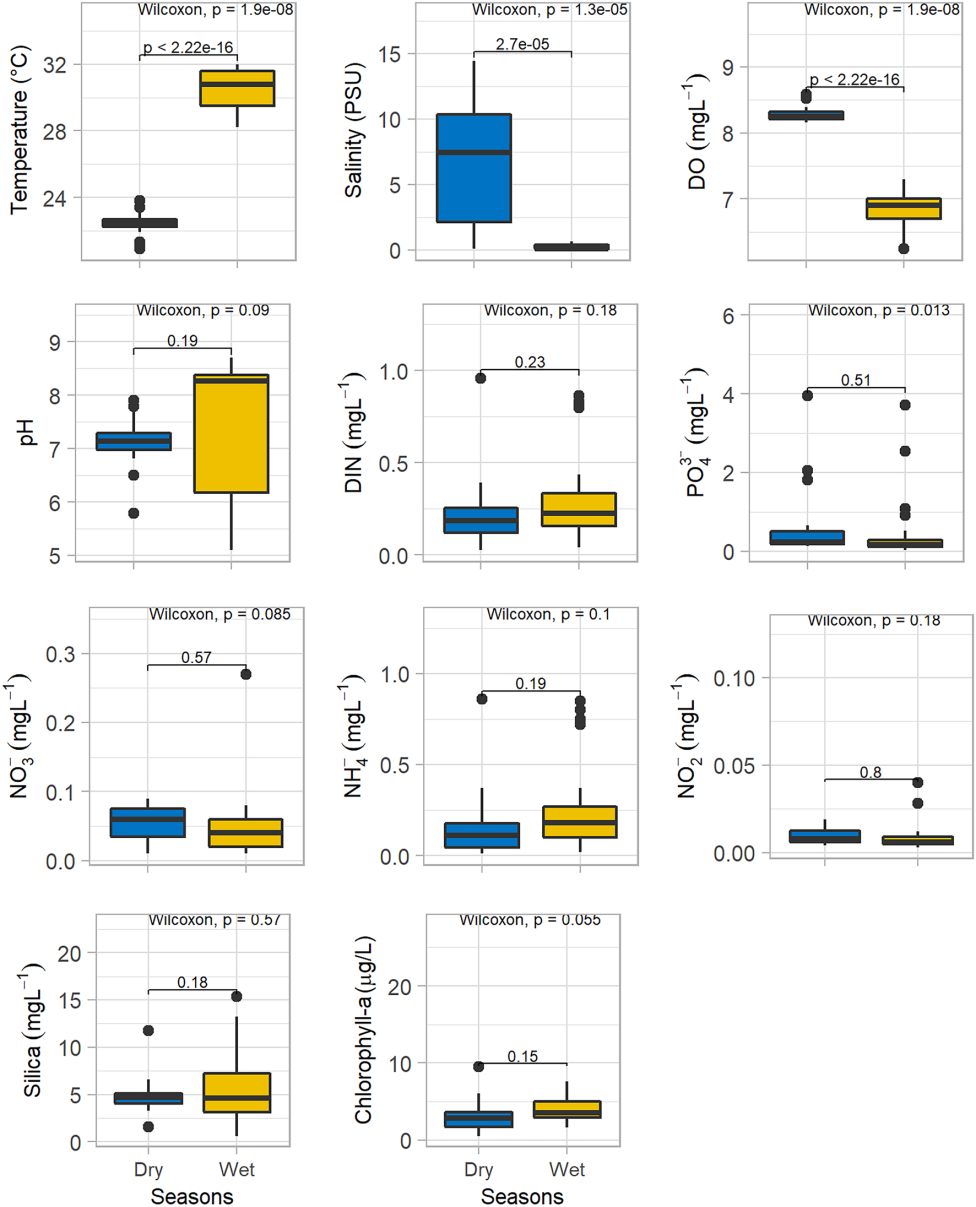
Temporal variations of major hydro-chemical parameter, dissolved inorganic nutrients and chlorophyll-a in the lower Meghna River estuary (UE = Upper estuary, ME = Middle estuary, LE = lower estuary, CK = Char Kukrimukri, HI = Hatiya Island). Note: the top, middle and bottom lines of the Box plot denote the upper quartiles, median and lower quartiles, respectively. The vertical line extending upward and downward denotes the range of data distribution. A data point located outside the whiskers of the box plot is called an outlier.

**Figure 5:**
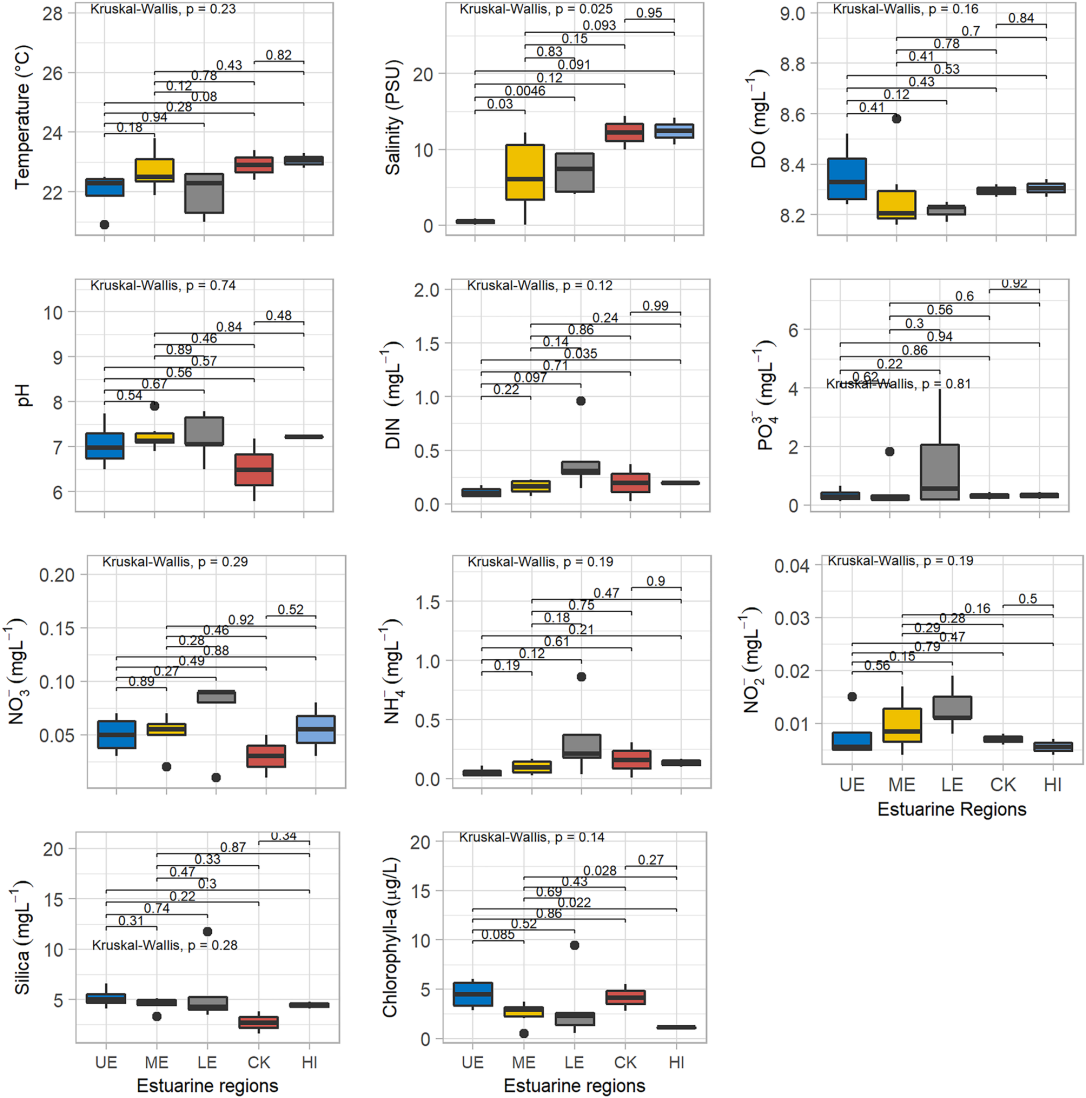
Spatial distributions of major hydro-chemical parameter, dissolved inorganic nutrients and chlorophyll-a in the lower Meghna River estuary during the dry season (UE = Upper estuary, ME = Middle estuary, LE = lower estuary, CK = Char Kukrimukri, HI = Hatiya Island). Note: the same for the Fig. 4.

**Figure 6:**
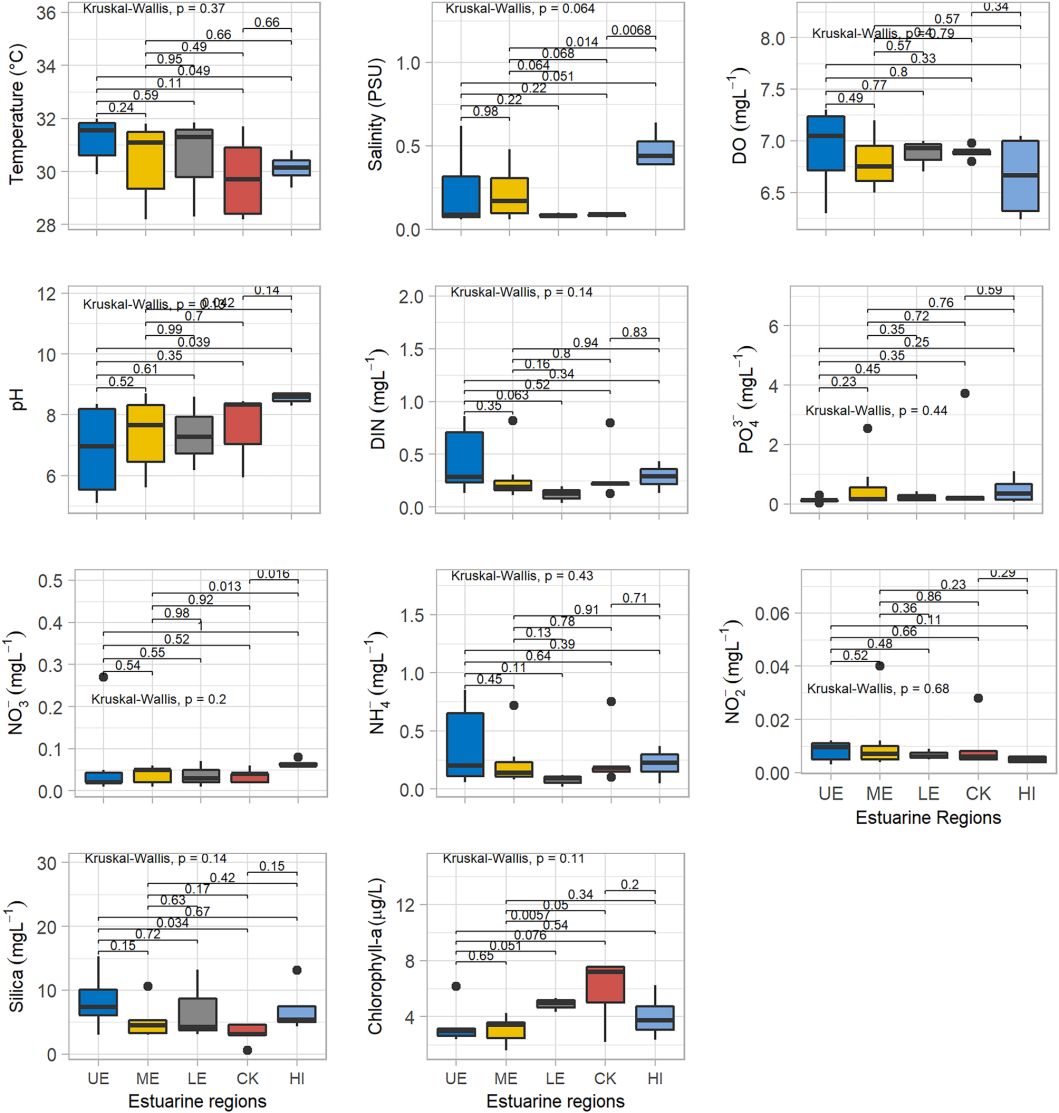
Spatial distributions of major hydro-chemical parameter, dissolved inorganic nutrients and chlorophyll-a in the lower Meghna River estuary during the wet season (UE = Upper estuary, ME = Middle estuary, LE = lower estuary, CK = Char Kukrimukri, HI = Hatiya Island). Note: the same for the Fig. 4.

**Figure 7:**
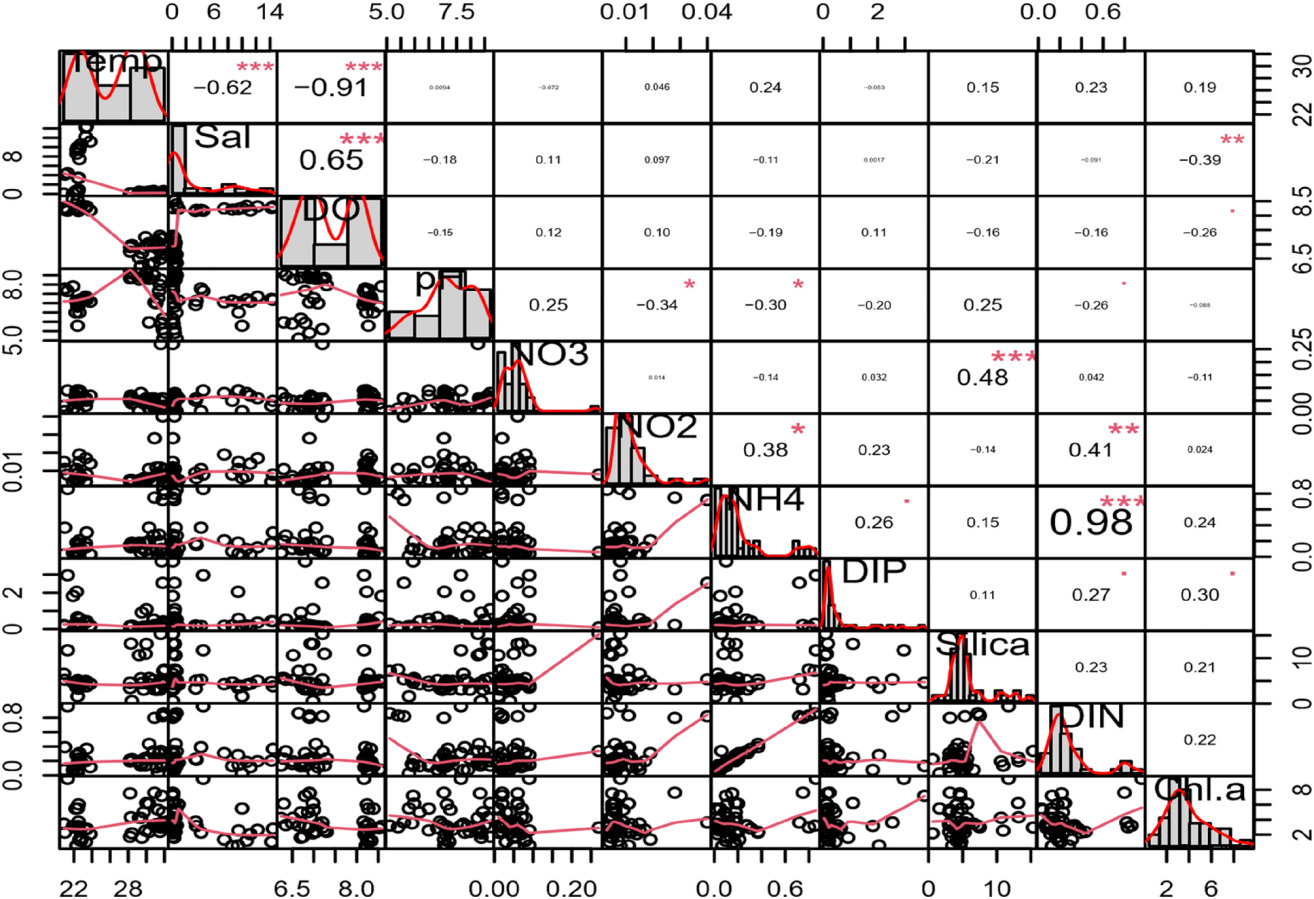
The correlation plot among the ten-water quality parameter. The values given around each axis are a range of individual parameter. The correlation coefficient (r) is indicated by a numeric value and the significance levels (p) is indicated by asterisk (* < 0.05, ** < 0.01, *** < 0.001).

Water quality parameter showed significant seasonal variations and insignificant spatial variations (Table 1; Figs. 4, 5, 6). To understand the spatial variations, the monitoring stations were arranged from the upstream to the downstream (Figs. 5, 6). Water temperature varied significantly between the dry (22.5 °C) and wet (30.8 °C) seasons. The median water temperature of 22.5 °C during the dry season was significantly different (p < 0.01) from that of the wet season (30.5 °C). The spatial concentrations of salinity and pH were generally low at the upstream stations (UE), gradually increasing at the mid (ME) and downstream (LE) stations (Table 1, Fig. 4). The lowest salinity of 0.9 PSU was observed during the wet months (August and October), and the highest salinity of 13.0 PSU was measured in the dry months (Figs. 2, 3, 4, 5 and 6). The mean salinity values differed significantly (p < 0.01) between the wet and dry seasons due to large variations in river discharge (Fig. 4). According to the salinity ranges^41,42^, an estuary can be classified into five Venice salinity classes viz. euhaline (salinity > 30 ﻿PSU), polyhaline (salinity 18–30 PSU), mesohaline (salinity 5–18 ﻿PSU), oligohaline (salinity 0.5–5 PSU) and freshwater system (salinity < 0.5 PSU). The MRE acts as freshwater system during the wet season on the basis of Venice salinity classes (Table 1). Significant (p < 0.01) spatiotemporal variation in pH were not found in the water samples (Fig. 4). pH showed an increasing trend from fresh to marine zone during the wet season (Table 1, Fig. 4)). The lowest value of 6.8 was found at the fresh zone (UE) and the highest value (8.6) at marine zone HI. The median values of pH were 7.1 during the dry season and 8.3 during the wet season. The DO concentration was 8.3 mg/l during the dry season and 6.9 mg/l during the wet season. The observed DO level during the dry season was significantly (p < 0.01) higher than the wet season (Fig. 3). It is assumed that higher values of DO were observed during the dry seasons due to higher photosynthetic activity with low turbidity, and lower values during the wet season due to oxidation of organic matter with high turbidity. In addition, more organic waste enters the estuarine waters during the wet season along with huge freshwater runoff. Significant differences were observed in temperature, salinity and DO between the wet and dry seasons (Fig. 4). Among these parameters, only the salinity showed significant spatial variation (Figs. 5, 6). In addition, salinity showed significant positive correlation with DO (p < 0.001, Fig. 7). Relevantly, salinity was negatively correlated with temperature (p < 0.001) and chlorophyll-a (p < 0.01).In addition, there was a distinct negative correlation between pH and NO_3_^−^/NH_4_^+^. Although DO was inversely correlated with chlorophyll-a, indicating that biological processes are not only the factor affecting DO in the estuary. Negative correlations between temperature and DO were highly significant (p < 0.001), and highly significant positive correlations was found between NH_4_^+^ and DIN (p < 0.001, Fig. 7).

**Table 1:** Median and standard errors of physical, chemical and biological parameters in different sections of the MRE during dry and wet seasons.

Parameter	Seasons	Mean values of water quality parameter of different sites					Median	Standard Errors					Median
		UE*	ME*	LE*	CK*	HI*		UE	ME	LE	CK	HI	
Temperature (°C)	Dry	22.0	22.7	22.0	22.9	23.1	22.5	0.4	0.3	0.3	0.5	0.3	0.17
	wet	31.2	30.4	30.5	29.8	30.1	30.8	0.4	0.5	1.1	0.7	0.3	0.25
Salinity (PSU)	Dry	0.50	6.5	7.0	12.2	12.4	7.45	0.2	2.0	1.2	2.2	1.8	1.14
	wet	0.22	0.22	0.08	0.09	0.48	0.1	0.10	0.06	0.01	0.01	0.06	0.04
DO (mg/l)	Dry	8.4	8.3	8.2	8.3	8.3	8.25	0.06	0.07	0.01	0.03	0.04	0.03
	wet	6.9	6.8	6.9	6.9	6.7	6.9	0.2	0.1	0.1	0.0	0.2	0.05
pH	Dry	7.1	7.3	7.2	6.5	7.2	7.14	0.27	0.14	0.23	0.70	0.01	0.14
	wet	6.8	7.4	7.3	7.6	8.6	8.26	0.6	0.5	0.7	0.5	0.1	0.30
NO_3_^−^ N (mg/l)	Dry	0.05	0.05	0.07	0.03	0.06	0.06	0.01	0.01	0.02	0.02	0.03	0.006
	wet	0.07	0.04	0.04	0.04	0.07	0.04	0.04	0.01	0.02	0.01	0.01	0.01
NO_2_^−^ N (mg/l)	Dry	0.008	0.01	0.013	0.007	0.005	0.008	0.002	0.002	0.002	0.001	0.001	0.001
	wet	0.008	0.012	0.007	0.01	0.005	0.006	0.002	0.005	0.001	0.004	0.001	0.002
NH_4_^+^ (mg/l)	Dry	0.06	0.10	0.33	0.16	0.14	0.16	0.02	0.02	0.14	0.15	0.04	0.04
	wet	0.37	0.23	0.08	0.27	0.22	0.25	0.15	0.09	0.03	0.12	0.07	0.05
PO_4_^3−^/DIP (mg/l)	Dry	0.34	0.49	1.39	0.31	0.33	0.34	0.11	0.27	0.73	0.13	0.12	0.18
	wet	0.13	0.59	0.23	0.89	0.46	0.46	0.04	0.34	0.11	0.71	0.23	0.17
DIN (mg/l)	Dry	0.11	0.16	0.42	0.20	0.20	0.18	0.02	0.03	0.14	0.17	0.01	0.05
	wet	0.44	0.28	0.12	0.32	0.29	0.29	0.13	0.09	0.05	0.12	0.06	0.05
DSi (mg/l)	Dry	5.19	4.53	5.75	2.71	4.46	4.79	0.51	0.27	1.52	1.09	0.33	0.44
	wet	8.3	5.0	6.9	3.2	7.1	5.99	1.8	1.0	3.2	0.7	2.0	0.75
Chlorophyll-a (µg/l)	Dry	4.46	2.54	3.24	4.15	1.11	3.15	0.77	0.47	1.60	1.35	0.07	0.51
	wet	3.4	3.0	4.9	5.9	4.0	4.10	0.6	0.4	0.3	1.0	0.8	0.36

#### Nutrients

Among the major nutrients, dissolved inorganic nitrogen (DIN) values ranged from 0.29 mg/l during the wet season to 0.18 mg/l during the dry season. During the wet season, high concentrations of NO_3_ -N were found at HI and UE areas (Table 1) due to inflow of nutrient-rich waters from the upstream. The NO_3_ -N concentrations were higher during the dry season. DIN and NH_4_^+^ showed higher concentrations during the wet season as well as higher values in estuarine and marine zones compared to the dry season (Table 1, Figs. 5, 6). The wet season was characterized by high DIN levels (Table 1). The NH_4_^+^ was the major inorganic form of DIN during both the wet and dry seasons (Table 1 and Fig. 4). The NH_4_^+^ concentrations attained the maximum percentage of 51.6 and 80.7% in the dry and wet seasons, respectively.

Dissolved inorganic phosphorus (DIP) values varied from 0.46 mg/l during the wet season to 0.34 mg/l during the dry season (Table 1). Higher values were observed at the Char Kukrimukri (CK) compared to other areas (Table 1). Significant differences were observed in the PO_4_-P between the wet and dry seasons (Fig. 4). DIP did not fluctuate significantly throughout the sampling station (Figs. 5, 6). DSi values were higher in the riverine zone (UE) than in the estuarine (LE) and marine zones (HI and CK). Dissolved silica (DSi) loadings in coastal water enhance the production of diatoms. This trend was found during the dry and wet seasons (Table 1, Figs. 5, 6). In the wet season, the DSi concentrations were generally higher (5.99 mg/l). In the lower portion (CK, HI) of the MRE, DSi values decreased to the lowest concentration of 3.1 mg/l where salinity values were higher. The dissolved silica (DSi) concentration did not differ significantly between the dry and wet seasons (Fig. 4). The distributions of silica did not show significant spatial variations (Figs. 5, 6). Nutrients were low in concentration, consistent with tropical conditions.

#### Chlorophyll-a (Chl-a)

Significant differences were observed in the chlorophyll-a between wet and dry seasons (Fig. 4). Mean chlorophyll-a concentrations were higher at the Char Kukrimukri area (CK), followed by the estuarine (UE, ME, LE) zone. The highest chlorophyll-a values were found at CK during the wet season. The vertical salinity distribution across the mouth of the lower Meghna River estuary also showed the westward outflowing of freshwater to the CK that induced chlorophyll-a production at CK zones of the MRE (Figs. 4, 5, 6). The highest chlorophyll-a values were also found at Mangalore coast in India in the wet season^8^. The Char Kukrimukri (CK) zone may be characterized as mesotrophic estuarine zone (LE, ME, UE) based on chlorophyll-a concentration^8,43^. Based on 80th percentiles values of chlorophyll-a^44^, an estuary can be classified into three states: Oligotrophic (Chlorophyll-a: 0–5 µg/l), mesotrophic (Chlorophyll-a: 5–20 µg/l) and eutrophic (Chlorophyll-a: 20 ~ −60 µg/l). The highest chlorophyll-a value was found during the wet season (Table 1, Fig. 4). The MRE can be classified as mesotrophic based on chlorophyll-a concentration. Chl-a values differed significantly between the wet (6.8 µg/l) and dry (5.6 µg/l) seasons (Table 2). However, Chl-a values showed a significant correlation with PO_4_-P (p < 0.05) and insignificant correlation with DIN (p > 0.05) (Fig. 7). A significant negative correlation was found between Chl-a and salinity (p < 0.01). The Chl-a variation showed an opposite pattern to salinity (Figs. 5, 6). In addition, chlorophyll-a also showed different spatial patterns between the two seasons (Table 1 and Fig. 4). The dry period was characterised by concentration at the upper portion (UE). During the rainy (wet) season, the largest chlorophyll a peak (5.9 µg/l) occurred in higher-salinity zone of the lower portion (Char Kukrimukri) of the MRE (Fig. 6).

**Table 2:** Indicator threshold values to classify the trophic status of the Meghna River estuary.

Indicator parameter	Seasons		Classification System				Methods	References
	Dry	Wet	Good*	Fair*	Poor*	Very poor*		
DIN (mg/l)	0.29	0.36	0 to < 0.1	≥ 0.1 but < 1	> 1	–	80th percentile^45^	Dodds^45^
DIP (mg/l)	0.59	0.45	0 to < 0.01	≥ 0.01 but < 0.1	> 0.1	–	80th percentile^45^	
Dissolved silica (mg/l)	5.1	8.1	> 5	> 2 but ≤ 5	> 0 but ≤ 2	0	10th percentile^46^	Bricker et al.^46^
Phytoplankton biomass (µg/l)	5.6	6.8	0 to ≤ 5	> 5 but ≤ 20	> 20 but ≤ 60	> 60	90th percentile^43^	Garmendia et al.^43^

*Good = Oligotrophic; Fair = Mesotrophic; Poor = Eutrophic; Very poor = Hypereutrophic; UE = Upper estuary, ME = Middle estuary, LE = lower estuary, CK = Char Kukrimukri, HI = Hatiya Island.

### Phytoplankton community composition and diversity in water

In this study, we encountered twenty-seven phytoplankton species belonging to Chlorophyta (green algae), Cyanobacteria (blue-green algae), Miozoa (Dinoflagellates) and Bacillariophyta (diatoms). Among these phyla, Chlorophyta was the most dominant class (Table 3). Two species of blue-green algae, *Oscillatoria* sp. and *Microcystis* sp., a diatom, *Lialoma* sp., and a green alga, *Pediastrum* sp. were found in all the sampling stations. *Spirogyra* sp. and *Oscillatoria* sp. dominated in upper (UE) and middle estuary (ME) during the dry season. Chlorophyta was the dominant group ranging from 36% during the wet season to 26% during the dry season (Table 3). In addition, Chlorophyta was the dominant group at all the sampling stations ranging from 27% (CK) to 35% (UE) during the wet season. In the dry season, phytoplankton density varied from 16.2 × 10^3^ to 94.1 × 10^3^ cells L^−1^, with the highest count observed in the upper estuarine region (UE). In contrast, in the wet season, phytoplankton density varied from 10.3 × 10^3^ to 215.1 × 10^3^ cells L^−1^, the highest count was observed in the Char Kukrimukri (CK) and Hatiya Island (HI) regions. Phytoplankton community structure was governed by *Spirogyra* sp. in upper estuary (UE) and *Pediastrum* sp. in the Char Kukrimukri (CK) and Hatiya Island (HI) regions during the wet season*.* In general, the Chlorophyta was the dominant phyla during both the dry and wet seasons (Table 3) when nitrogen and phosphorus concentrations were optimal for their abundance. In contrast, Bacillariophyta was the second dominant phyla in the wet season under oligohaline (salinity < 0.5 PSU) condition. However, Bacillariophyta succeeded Chlorophyta in the dry season and consequently depleted dissolved silica (Fig. 4). Thus, the seasonal succession of phytoplankton between Bacillariophyta and Chlorophyta occurred in the dry season in the MRE. Simpsons Reciprocal Index is directly proportionate to species diversity (Table 4). The highest diversity was found in the MRE during both the dry (D < 0.16, 1/D > 6.0) and wet (D < 0.19, 1/D > 5.2) seasons.

**Table 3:** Phytoplankton genera observed in the hilsa gut and water.

Phytoplankton division	Seasons	Genus		Percent (%) contribution	
		Hilsa gut	Water	Hilsa gut	Water
Bacillariophyta (diatoms)	*Dry*	*Synedra* sp., *Coscinodiscus* sp., *Pleorosigma* sp., *Triceratium* sp., *Navicula* sp., *Fragilaria* sp., *Lioloma* sp., *Ditylum* sp., *Odontella* sp., *Synedra* sp*.* and *Gomphonema* sp.	*Coscinodiscus* sp., *Lioloma* sp., *Asterionella* sp., *Pleorosigma* sp., *Chaetoceros* sp., *Proboscia* sp., *Ditylum* sp., *Cerataulina* sp., *Fragilaria* sp., *Melosira* sp., *Asteromphalus* sp., *Triceratium* sp. and *Nitzchia* sp.	21.6	32
	*Wet*	*Coscinodiscus* sp., *Melosira* sp., *Synedra* sp., *Fragilaria* sp., *Asterionella* sp., *Odontella* sp., *Diatoma* sp., *Navicula* sp., *Surirella* sp., *Nitzchia* sp*.* and *Lioloma* sp*.*	*Coscinodiscus* sp.,* Lioloma* sp.,* Pleorosigma* sp., *Cyclotella* sp., *Fragilaria* sp., *Asterionella* sp., *Odontella* sp., *Licmophora* sp., *Synedra* sp., *Thalassonema* sp., *Surirella* sp., *Triceratium* sp., *Melosira* sp*.* and *Nitzchia* sp*.*	23.5	27
Chlorophyta (green algae)	*Dry*	*Oedogonium* sp., *Spirogyra* sp., *Pediastrum* sp., *Muogeotia* sp.,* Ulothrix* sp., *Microspora* sp., *Volvox* sp., *Tetraedron* sp., *Chlorella* sp., *Muogeotia* sp., *Zygnema* sp., *Closterium* sp*.* and *Stichococcus* sp*.*	*Hydrodictyon* sp., *Muogeotia* sp., *Microspora* sp., *Chlorella* sp., *Volvox* sp., *Ulothrix* sp., *Merismopedium* sp., *Uroglena* sp., *Closterium* sp., *Pediastrum* sp., *Oedogonium* sp., *Spirogyra* sp. and *Tetraedron* sp.	45.8	26
	*Wet*	*Muogeotia* sp., *Spirogyra* sp., *Zygnema* sp., *Ulothrix* sp., *Pediastrum* sp., *Phytocoris* sp., *Cladophora* sp*.* and *Arthrospira* sp*.*	*Hydrodictyon* sp., *Pediastrum* sp., *Oedogonium* sp., *Muogeotia* sp., *Phytoconis* sp., *Microspora* sp., *Volvox* sp., *Arthrospira* sp., Zygnema sp., *Spirogyra* sp. and *Tetraedron* sp.	61.5	36
Cyanobacteria (blue-green algae)	*Dry*	*Oscillatoria* sp*.*, *Chroococcus* sp*.*, *Microcystis* sp*.*, *Aphanizomenon* sp*.*, *Gomphosphaeria* sp*.*, *Anabaena* sp*.* and M*erismopedium* sp*.*	*Microcystis* sp., *Gomphosphaeria* sp., *Oscillatoria* sp., *Aphanizomenon* sp., *Lyngbya* sp. and *Rivularia* sp.	14.08	16
	*Wet*	*Gomphosphaeria* sp.,* Microcystis* sp., *Oscillatoria* sp., *Gleocapsa* sp*.* and *Anabena* sp*.*	*Microcystis* sp., *Anabaena* sp., *Gomphosphaeria* sp., *Oscillatoria* sp.	12	14
Xanthophyta	*Dry*	*Botrydium* sp*.*	–	2.66	
	*Wet*	–	–	–	
Euglenophyta	*Dry*		*Euglena* sp.		3
	*Wet*	*Euglena* sp.	*Euglena* sp.	1	4
Miozoa	*Dry*	–	*Ceratium* sp.		9
	*Wet*	–	*Ceratium* sp. and *Detonula* sp.		4

**Table 4:** Phytoplankton diversity index values during the dry and wet seasons.

Diversity indices	Seasons	UE*	ME*	LE*	HI*	CK*	Median
Simpsons Index (D)	Dry	0.17	0.13	0.16	0.37	0.14	0.16
	Wet	0.18	0.13	0.28	0.19	0.20	0.19
Simpsons Reciprocal Index (1/D)	Dry	5.80	7.46	6.01	2.67	6.98	6.01
	Wet	5.63	7.75	3.53	5.21	5.12	5.21

*UE = Upper estuary, ME = Middle estuary, LE = lower estuary, CK = Char Kukrimukri, HI = Hatiya Island.

### Phytoplankton composition in the hilsa fish gut as the source of polyunsaturated fatty acids

Phytoplankton are producers and major suppliers of polyunsaturated fatty acids in estuary and coastal ecosystems, and are important for the function and quality of the entire coastal food web. A meta-analysis of more than 160 fatty acid profiles from seven marine phytoplankton phyla reveals a highly class-specific PUFA production by marine phytoplankton^19^. Among them, the highest amount of PUFA is found in Chlorophyta, which accounts for 60% of the total fatty acids^19^. The lowest PUFA is found in Cyanobacteria and diatoms (26 and 28% respectively). In this study, a mixed population of Chlorophyta (45.8 and 61.5% during the dry and wet seasons, respectively), diatoms (21.6 and 23.5% in the dry and wet seasons, respectively) and Cyanobacteria (14.1 and 12% in the dry and wet seasons, respectively) contributed approximately 89.3% to the composition of gut phytoplankton in the hilsa fish of the MRE. The phytoplankton composition indicates that Chlorophyta was the principal source of PUFA for hilsa fish, followed by diatoms and Cyanobacteria.

## Discussion

### Seasonal and spatial water quality

Variation of water quality is represented by sampling points (spatial effect) and sampling months (seasonal effect)^47^. Among water quality parameter, dissolved oxygen is an important indicator^48^. Decreased DO levels during the rainy (wet) season are related to the amount of oxygen consuming compounds entering from nearby industrial or agricultural areas through estuary river runoff. Low salinity during the rainy (wet) season was due to the outflow of fresh water. In contrast, during the dry season, the upper region (UE) remained oligohaline and the remaining sections (ME, LE, CK and HI) become mesohaline^41^. It is interesting that hilsa shad, an anadromous fish, can tolerate a wide range of salinity as it travels to different areas to find the best salinity to suit the different stages of its life cycle^49–51^. For example, hilsa prefers freshwater for spawning and nursing of the juveniles, the young (pre-adult called jatka) ones need estuarine and coastal water and the adult requires high saline marine water. However, the ideal salinity for the spawning and nursery activities of hilsa is < 0.1 PSU^14,15^. Thus, the entire MRE is suitable for hilsa spawning and nursery habitat during the wet season (Figs. 2, 4, 5, 6) when hilsa migrate to the MRE for spawning^52^. In contrast, only the upper region (above Ilisha ghat, UE) of the MRE acts as suitable hilsa spawning and nursery habitat during the dry season as hilsa fish breeds all the year round^40^.

The nutrient dynamics and primary productivity (indicated by Chl-a) of the MRE were strongly influenced by the seasonal fluctuations in precipitation. The MRE area is characterized by numerous distributaries. Annual rainfall is characterized by a typical monsoon with a well-defined maximum and minimum period, facilitating nutrient outflow from the Ganges–Brahmaputra-Meghna River system^53^. Similar seasonal patterns have been identified on the Brazilian coast^54^ and several other tropical estuarine areas^54,55^.

**Figure 8:**
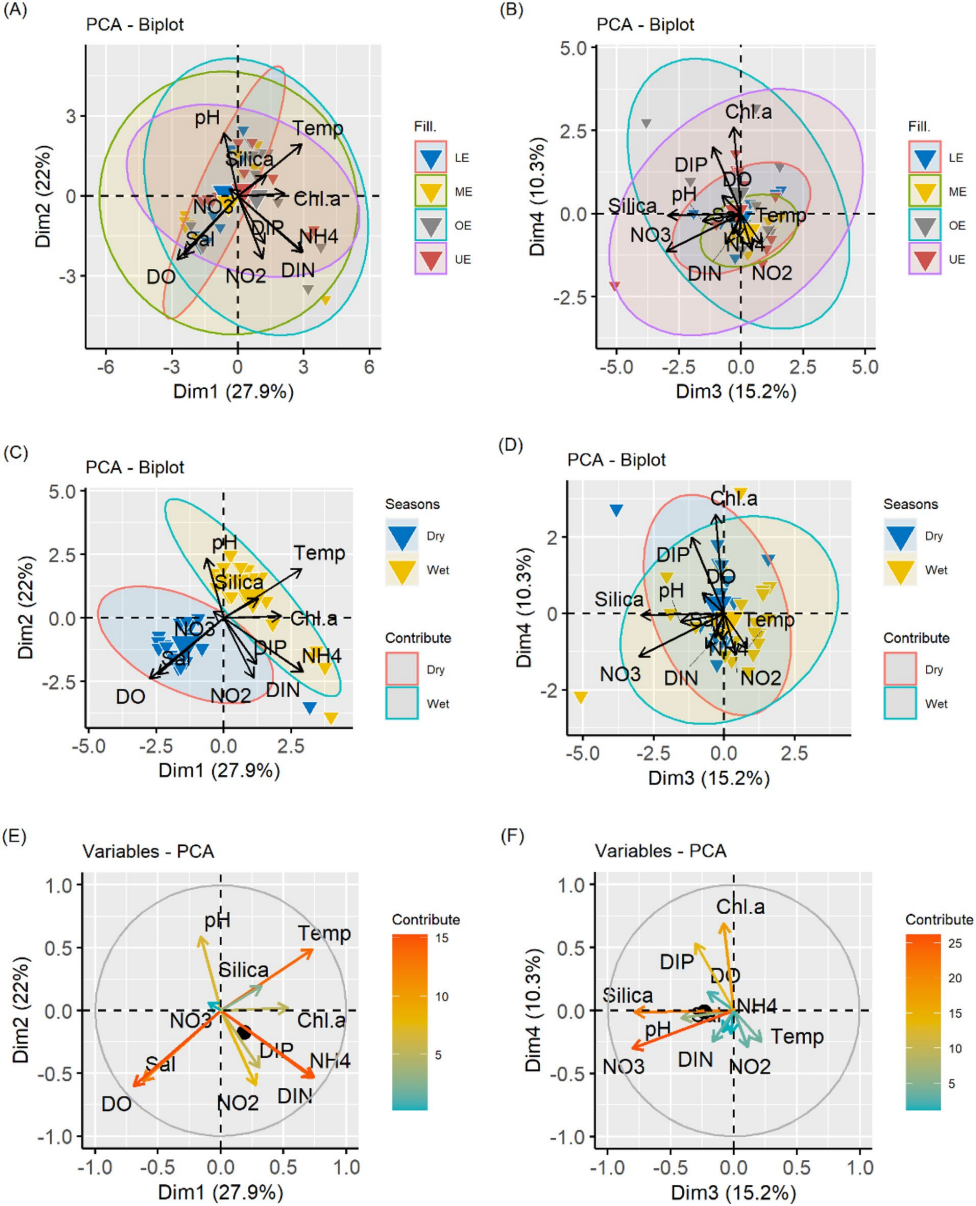
Factorial plan of the first, second, third and fourth axes of the principal component analysis (PCA) for the spatial (**A**, **B**), temporal (**C**, **D**, **E**, **F**) environmental parameter of the lower Meghna River estuary (UE = Upper estuary, ME = Middle estuary, LE = lower estuary, CK = Char Kukrimukri, HI = Hatiya Island).

The input of large amounts of anthropogenic DIN (mainly NO_3_^−^ and NH_4_^+^), the form of nitrogen that phytoplankton use directly, alters the estuarine and coastal marine ecosystem structure and function (species composition, abundance, distribution, and production, species diversity, and dynamics) of many coastal ecosystems^56^. The effects of different sources on the nutrient dynamics and chlorophyll-a distribution in the MRE can be explained by the results of a principal component analysis (PCA) performed separately in the dry and wet seasons. The first variable factor (DIM1) explained 27.9% of the variations in water quality fluctuations and contained the most information (Fig. 8). DIM1 clearly showed a clear positive correlation with temperature, DIN and NH_4_^+^ (Fig. 8). Inorganic source of dissolved nitrogen in the rainy (wet) season, might be due to human activity, and can be interpreted as an effect from non-point sources, such as freshwater discharges. Inorganic nitrogen fertilizer use, and nitrogen fixation in agricultural systems comprise the largest sources of the N inputs^57^. Seasonally, the nutrient concentrations in freshwater may be even lower than those found in the coastal ocean, which lead to complex management problems^58^. Although, the DIN export in rivers to the coastal ocean nearly doubled over the past four decades of the twentieth century globally, DIN species are in low concentrations in tropical coastal water^59^. DIN variation among Indian coastal ecosystems can be caused by differences in the freshwater input pulse^59^. Ammonium is the dominant N form in estuarine and coastal areas except in polluted estuaries and eutrophic and coastal upwelling regions^59,60^. This scenario is true for most of the Indian mangroves; however, some mangroves have nitrate as the major DIN pool. The dominant DIN pool varies depending on source, processing, and other environmental factors. High river discharge and runoff plays a key role in bringing these nutrients (DIN and NH_4_^+^) to this system during the wet season. Temporally, the impact of DIN and NH_4_^+^ on the phytoplankton biomass production were greater in the wet season than in the dry season (Fig. 8). In addition, DIM1 had a moderate correlation with chlorophyll-a. Therefore, DIM1 should mainly be interpreted as one kind of dissolved inorganic nitrogen source that is mainly affected by a non-point source.

Salinity and temperature are key environmental traits for the physical structure of estuaries, those influence phytoplankton (chlorophyll a is proxy for that) communities^61^. DIM2, which accounts for 22.0% of the total variance, had a negative correlation of chlorophyll a with dissolved oxygen and salinity, and a positive correlation of chlorophyll a with temperature (Fig. 8). Dissolved oxygen levels increase with freshwater runoff and decrease with rise in temperature^62^. DIM2 can be represented as a physicochemical source due to the natural changes in the aquatic environment and the ionic properties of the water body.

Phytoplankton abundance in coastal waters may be due to fluctuations in the essential nutrients (such as nitrate, phosphate and silicate), from either an upwelling or run-off^61^. In coastal areas, nitrate is the dominant N form and preferred by diatoms^63^. The uptake of nitrate in coastal areas can be high and may be reduced if ammonium is available^64^. The ammonium concentration that suppresses nitrate uptake varies greatly and strongly depends on phytoplankton species. For benthic diatoms, the uptake of other N compounds is suppressed when NH_4_^+^ is present^65^. Silicate is an important factor for the growth of diatoms and leading to the shifts of different phytoplankton groups^66^. DIM3 and DIM4, which accounts for 25.5% of the total variance, were weighted on dissolved silica, NO_3_^−^, PO_4_^3−^ and chlorophyll-a (Fig. 8). The correlation matrix showed that DSi, NO_3_^−^ and PO_4_^3−^ correlates with chlorophyll-a during the wet season (Fig. 8). Therefore, DIM3 and DIM4 represents the natural source of dissolved inorganic nutrients, primarily reflecting the natural changes in the aquatic environment, and the growth of phytoplankton^67^. The PCA plot shows a seasonal gradient observed at the sampling site, forming two different groups (Fig. 8). This analysis also showed biochemical processes that occur in the MRE waters during the dry and wet seasons.

### Phytoplankton community

Bioavailable nitrogen is the principal limiting nutrient in estuaries for phytoplankton^68,69^. A mixed population of Chlorophyta, diatoms and blue-green algae contributed approximately 77% to the composition of wet season phytoplankton in the MRE due to favourable temperature and nutrients (Table 3) and 74% during the dry season phytoplankton. The Ganges, Brahmaputra and Meghna are the most important contributors to DIN during the wet season and green algae proliferate due to relatively high nitrogen levels. Dissolved inorganic nitrogen (nitrates, nitrites and ammonia) come mainly from nearby catchment areas and enter into estuaries via adjacent rivers and surface runoff^70^. In addition, denitrification, tidal flushing and hydraulic loading influence nitrogen loads within the estuary^71^. In contrast, the bioavailability of phosphorous (P) depends on the river load, the degradation of organic matter, and the ingress of coastal waters into the adjacent estuary during the high tide^72^, which becomes more frequent during the wet season.

The nutrient in freshwater causing most blue-green blooms in particular is phosphorus^61^. In addition, Cyanobacteria prefer nitrogen in the form of ammonia and nitrate^61^. Cyanobacterial abundance was driven by an increased presence of nutrients (ammonia, nitrate and phosphorus) in the MRE during the wet season (Fig. 6 and Table 1) because most phytoplankton, except some species belonging to Cyanobacteria cannot fix atmospheric nitrogen^61^. Salinity is an additional environmental factor that can have some impact on the algal abundance in the freshwater system^61^. Information is scarce on the salinity tolerances of most freshwater phytoplankton species. Two species of Cyanobacteria such as *Anabaena* sp. and *Microcystis* sp., have a salt tolerances of up to 5–6 PSU before they are killed off by salinity^73^. In contrast, green algae are the most abundant and diverse of all freshwater algae^61^. The green algae are primarily a freshwater group, with approximately 62% of representatives occurring in both the dry and wet seasons (Fig. 6).

In the MRE, diatoms predominate after a decrease in river flow during the dry season, and a mixed diatom populations contribute approximately 59% to the composition of phytoplankton in the dry season (Fig. 6). Such type of pattern has also been found in the Meuse River, Belgium^74^ and in the Thames River, UK^8^. However, the factors that regulate seasonal periodicity of phytoplankton in estuaries are less well documented and not yet fully understood than those in lakes^8^. The MRE provides the suitable conditions for the proliferation of diatoms and green algae, the main components of the phytoplankton. Silica must be considered as the first regulator of diatom development^8^. In the MRE, favourable hydrological conditions are met after the decrease of discharge in the dry season, as a consequence a high level of diatom abundance occurred during the dry season that depleted silica (Figs. 4, 5, 6). Silica content clearly affects the maximum diatom biomass that reaches during the dry season.

During the dry season, diatoms were the dominant group which was replaced by blue green algae (Cyanobacteria) during the wet season. Temperature above 25 °C provides suitable environments for blue-green algal growth rates. In addition, under lower light dose and stronger light fluctuations, net growth rate of Cyanobacteria tends to decrease^76^. Blue-green algae developed quickly in nutrient rich (high in phosphorus) environments with favorable temperature during the wet season^61^. Salinity is another environmental factor that may have some effect on algal presence in fresh water system^73^. Some blue-green algal species have been found to have salt tolerances of up to 5–6 PSU before they are killed off by salinity^61^. The concentration of silicates is essential for the growth of silicified organism’s diatoms^77,78^. Increasing silica inputs allowed the diatoms to grow at a higher level, leading to decrease in silica level in the water column. Conversely, drop in major nutrient (SiO_4_) levels could be observed during the dry season, when diatom flourished and that deplete silica concentration during the dry season^8^. During the wet season, a strong negative correlation was observed between the diatoms and concentration of silica, as diatoms need silica in addition to phosphate and nitrate to build their frustules. Silica is influencing negatively on the growth of diatoms as they consume silica in cell wall synthesis^77^.

### Polyunsaturated fatty acids (PUFA) content in the major phytoplankton groups

Phytoplankton (microalgae) is principal source of ω3 and ω6 PUFA^19^. Meta-analyses clearly show that some classes of phytoplankton are a better source of essential PUFAs than others^19^. Studies have shown that crustaceans and fish cannot easily biosynthesize ω3 and ω6 PUFAs^19^. These fatty acids have to be obtained from their diet available in the food web. The highest levels of PUFAs are found in green algae (approximately 60% of the total fatty acid)^19^ and the lowest levels of PUFA are found in blue-green algae (26%) and diatoms (28%). Therefore, the focus was given to the composition of the major phytoplankton group in both water and hilsa fish gut of the MRE. In this study, a mixed population of green algae (53.6%), blue-green algae (13%) and diatoms (22.6%) contributed approximately 89.2% to the composition of gut phytoplankton in the hilsa fish of the MRE. Hilsa fish was selected because it is the national fish of Bangladesh and is an important food fish, rich in ω3 and ω6 polyunsatured fatty acids, eicosapentaenoic acid (EPA) and docosahexaenoic acid (DHA)^79^. Fish or fish oil contains omega-3 PUFAs, e.g., DHA and EPA, which are beneficial to human health and reduce the risk of coronary heart diseases^80^. In addition, the ω3/ω6 and DHA/EPA proportions are high in diatoms^19^. The contribution of EPA and DHA to the mass of PUFAs differ in both proportion and quantity. Hilsa fish cannot synthesize these essential nutrients that must be obtained from the diet, especially via phytoplankton. Hilsa is omnivorous and mainly eats phytoplankton^81^. Higher levels of lipids and fatty acids in fish muscle, primarily DHA, are due to the high levels of lipids and DHA in the feed.

### Electivity index

**Figure 9:**
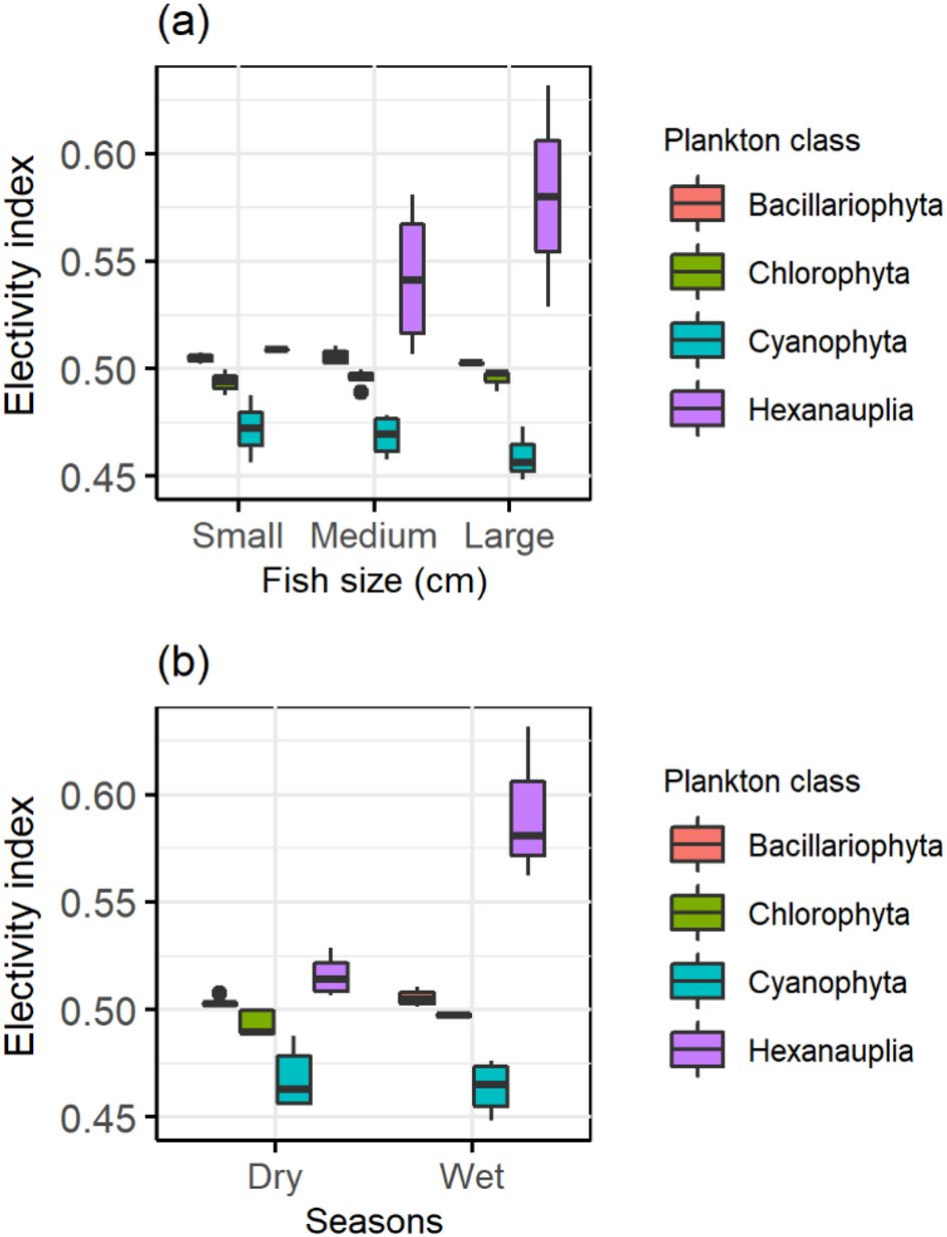
Electivity indices for (**a**) different size (small:10–20 cm, medium: 20–30 cm, large: 30–35 cm) of hilsa fish consuming (**b**) four abundant prey taxa (major group of phytoplankton) in the dry and wet seasons.

By comparing the relative quantity of a possible prey item with its relative predominance in a predator's diet, electivity indices summarize the findings of field-based feeding research. The numbers of distinct prey taxa found in water samples and the guts of hilsa fish were used to calculate a new electivity index based on odds ratios^28^. The electivity index value of 0.5 for the Chlorophyta and Bacillariophyta indicates the same prey sample in the water and in the hilsa fish gut (Fig. 9). Cyanobacteria is the third most preferable prey to hilsa fish following the hexanauplia (Fig. 9).

## Conclusion

We explored the ecological understanding of the seasonal periodicity of phytoplankton in the MRE with its changing hydrological conditions. No significant spatial variations were observed in the water quality parameters except salinity. Considering the salinity distribution, the entire MRE is a suitable hilsa spawning and nursery ground during the wet season and only the upper MRE (upstream of Ilisha ghat) can act as spawning ground during the dry season. The results of the multivariate analysis revealed two distinct groups for the dry and rainy seasons for the water quality criteria. The multivariate analysis explained 75.4% variability of seven physicochemical parameters that caused seasonal variations of three major groups of phytoplankton. The most relevant driving factors were dissolved oxygen, salinity, temperature, and DIN (nitrate, nitrite and ammonia). These variabilities in physicochemical parameters and dissolved inorganic nutrients caused seasonal variations in two major groups of phytoplankton. Peak abundance of green algae occurred in nitrogen and phosphorus-rich environment during the wet season. The diatoms were dominant during the dry season that severely depleted dissolved silica. Thus, phytoplankton diversity showed the potential link to seasonal changes of hydro-chemical parameters and phytoplankton development that was invariably initiated by the decrease of river discharge in the dry season. In addition, the green algae and diatoms were the major phytoplanktonic food for hilsa fish in the MRE food web as well as major source for PUFAs as higher percentage of green algae and diatoms were found in the hilsa fish gut.

## Data Availability

All data used to support the findings of the study are available from the corresponding author upon request.
